# StructNet-DDI: Molecular Structure Characterization-Based ResNet for Prediction of Drug–Drug Interactions

**DOI:** 10.3390/molecules29204829

**Published:** 2024-10-12

**Authors:** Jihong Wang, Xiaodan Wang, Yuyao Pang

**Affiliations:** 1 School of Computer, Guangdong University of Education, Guangzhou 510310, China; 2 School of Pharmaceutical Chemistry and Chemical Engineering, Guangdong Pharmaceutical University, Zhongshan 528458, China

**Keywords:** drug–drug interactions, SMILES, chemical structures, fingerprints, ResNet18

## Abstract

This study introduces a deep learning framework based on SMILES representations of chemical structures to predict drug–drug interactions (DDIs). The model extracts Morgan fingerprints and key molecular descriptors, transforming them into raw graphical features for input into a modified ResNet18 architecture. The deep residual network, enhanced with regularization techniques, efficiently addresses training issues such as gradient vanishing and exploding, resulting in superior predictive performance. Experimental results show that StructNet-DDI achieved an AUC of 99.7%, an accuracy of 94.4%, and an AUPR of 99.9%, demonstrating the model’s effectiveness and reliability. These findings highlight that StructNet-DDI can effectively extract crucial features from molecular structures, offering a simple yet robust tool for DDI prediction.

## 1. Introduction

In the treatment of complex diseases, combination therapy is a common and necessary approach [1,2]. However, when patients take multiple drugs simultaneously, the interactions between drugs may either enhance or diminish their effects and, in some cases, lead to adverse drug reactions [3], increasing patient morbidity and mortality [4]. These interactions are known as drug–drug interactions (DDIs). Beneficial synergistic drug interactions can improve efficacy and reduce side effects, whereas harmful DDIs may decrease efficacy and increase toxicity [5]. Therefore, identifying DDIs is crucial for enhancing treatment outcomes and ensuring patient safety. However, large-scale experimental studies of DDIs in the laboratory face challenges such as high costs and long durations.

In recent years, with the rapid development of computer technology, numerous machine learning- [6,7,8] and deep learning-based methods [9,10,11] have been proposed for DDI prediction. These methods are not only faster and more efficient but also help reduce unexpected drug interactions, lower drug development costs, and optimize the drug design process. As algorithmic techniques advance, DDI prediction methods have become increasingly diverse. Among these existing methods, most rely on the chemical [12], biological [13], and phenotypic [14] characteristics of drugs for feature extraction and model construction to achieve DDI prediction.

Li et al. [15] developed a probabilistic ensemble approach to construct a DDI prediction model using the molecular and pharmacological features of drugs, achieving an accuracy of up to 95%. Le et al. [16] constructed a framework called HAINI, using SMILES strings combined with CYP450-based interaction features to predict DDIs among histamine antagonists. These models, based on classical machine learning methods such as Random Forest (RF), Logistic Regression (LR), and XGBoost, outperformed those in previous studies, with the best accuracy being 0.788. Sun et al. [10] proposed a chemical substructure representation framework (CASTER) for DDI prediction based on drug chemical structures. CASTER employs sequential pattern mining to decompose the SMILES structures of drugs into substructures, smaller substructures, and atoms. An autoencoder module is then used to extract features from drug pairs and embed them into a latent space, which allows for better generalization to novel drug pairs. Nyamabo et al. [12] considered the chemical bonds in drug molecules as control gates and subvalent bonds as fracture operations, decomposing drug structures into substructures of varying sizes. They then used a co-attention layer to capture the interactions between each pair of substructures, assigning different weights to different types of drug interactions. Experimental results showed that their model achieved a DDI prediction accuracy of 98.46%. As indicated by these results and various literature reports [17], the chemical structures of drugs are a crucial factor in DDI prediction.

Existing DDI prediction methods often focus on drug similarity features, adverse effects, or drug-specific properties, as well as network relational associations. Although these approaches have proven effective, extensive research indicates that chemical attributes, particularly chemical structures such as SMILES representations, are crucial and cannot be overlooked in DDI prediction. Moreover, DDI prediction models based on the chemical attributes of drugs typically offer better interpretability.

However, since a molecule can be represented by multiple SMILES structures, this diversity presents challenges for model learning. To address these challenges, this paper proposes a StructNet-DDI model based on ResNet18 that utilizes the chemical structure characteristics, pharmacokinetics, and physicochemical properties of drug molecules. Experimental results demonstrate that this model, while simple, effectively highlights the importance and interpretability of molecular features in DDI prediction and achieves excellent performance.

## 2. Related Work

Early DDI prediction models primarily relied on processing the chemical, biological, and phenotypic characteristics of drugs as textual data. However, with the advent of deep learning breakthroughs in handling graphical data, graph-based data and graph structural models have gradually been utilized for DDI prediction [18,19,20]. To address the issue of traditional DDI prediction methods neglecting the positional information of atomic nuclei and edges in spatial structures, Wang et al. [21] proposed a lightweight drug interaction prediction method based on self-attention mechanisms. This method uses the 2D structures of drugs as input and encodes molecular graphs with four features related to spatial information, yielding excellent predictive results. Jiang et al. [22] introduced a DDI prediction method called RaGSECo based on relational graph structural embedding and contrastive learning. RaGSECo employs a cross-view contrastive mechanism, leveraging the latent correlations and co-supervision between drug pairs to enhance drug pair representation learning, resulting in significant improvements in DDI prediction.

Many existing DDI prediction methods focus on drug similarity features, adverse reactions, or side effects, as well as network relationship correlations [23,24]. Although these methods have proven effective, substantial research suggests that chemical properties, especially chemical structures like SMILES, are critical and indispensable features in DDI prediction. Moreover, DDI prediction models based on the chemical attributes of drugs offer clearer interpretability [25]. However, a single molecule can be represented by multiple SMILES structures, posing challenges for model learning [26,27].

Additionally, most existing molecular structure features are primarily textual, making it difficult for models to comprehend the relationship between SMILES text and the actual molecular structure [28]. Therefore, focusing on SMILES structures and using graphical approaches to establish associations among chemical structures, physicochemical properties, pharmacokinetics, and chemical bonds may enable more comprehensive DDI predictions. Yi et al. [29] proposed a DDI prediction model based on graph convolutional networks, emphasizing the chemical structure as the main feature, and the results demonstrated strong model performance.

This study explored molecular features and the SMILES graph structure separately. A promising direction would be the organic integration of these two representations, leveraging the powerful residual learning capabilities of ResNet18 to further enhance representation learning. Such a combination is expected to yield superior results, as it would not only extract deeper information from molecular features but also effectively capture the complex relationships within molecular structures. Therefore, integrating molecular features with SMILES graph structures through advanced residual learning presents a valuable area for further research.

## 3. Results and Analysis

### 3.1. Ablation Experiment

**Figure 1:**
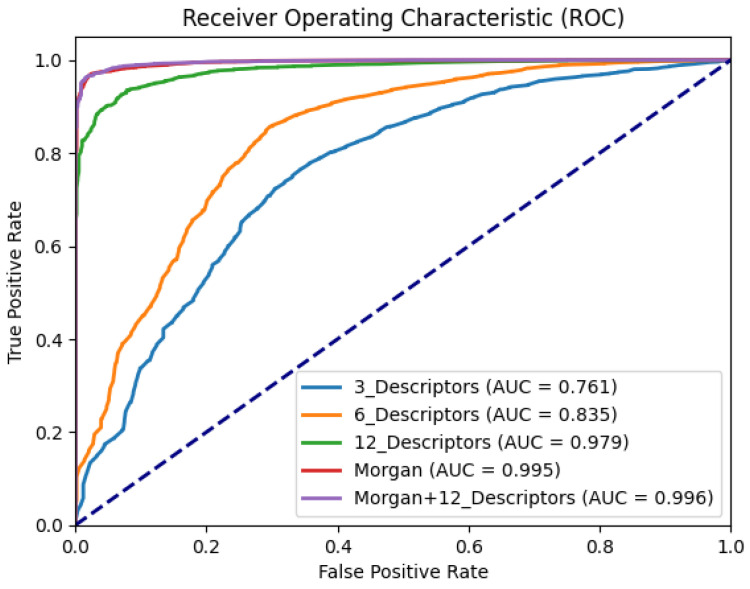
ROC curve of feature ablation experiment.

To better analyze the impact of drug molecular structural features on DDI prediction, we conducted ablation experiments on different features of StructNet-DDI. Five unique feature processing strategies were implemented in an ablation experiment. The results of these experiments are presented in Table 1 and Figure 1. Specifically, the ablation experiments involved the following five feature combinations:

1. 3_Descriptors: This experiment used only three basic molecular descriptors (molecular weight, logP value, and relative molecular mass) to assess the contribution of fundamental descriptors to DDI prediction.
2. 6_Descriptors: Building on the basic descriptors, the set was expanded to six descriptors (adding the number of rotatable bonds, the number of hydrogen bond donors, and the number of hydrogen bond acceptors) to evaluate the impact of a more enriched descriptor set on model performance.
3. 12_Descriptors: Further expanding to 12 molecular descriptors (adding the number of aromatic rings, the proportion of sp3-hybridized carbons, the number of nitrogen atoms, the number of oxygen atoms, the Fereyberling index, the topological polar surface area, and the number of free radicals), this experiment investigated the effect of the most comprehensive descriptor set on DDI prediction.
4. Morgan: This experiment independently used Morgan fingerprints to analyze its performance as a standalone feature, assessing the contribution of fingerprint features to DDI prediction.
5. Morgan + 12_Descriptors: Morgan fingerprints were systematically combined with the 12 molecular descriptors to examine whether this combined feature strategy could further improve model prediction performance.

These ablation experiments allowed us to identify the impact of different molecular structural features on DDI prediction, helping to optimize feature selection for achieving higher prediction accuracy and reliability.

The poor performance of models using a small combination of descriptors (such as three or six descriptors) can likely be attributed to the lack of sufficiently rich and comprehensive feature information, which hampers the model’s ability to effectively capture the characteristics and patterns of compounds [30]. However, when the number of molecular descriptors is expanded to 12, the set includes not only basic physicochemical properties and secondary bond information in molecular structures (such as hydrogen bonds and van der Waals forces) but also molecular orbital properties and pharmacokinetic characteristics. DDIs are primarily categorized into pharmacokinetics and pharmacodynamics [31,32]. Therefore, from a pharmacokinetic perspective, these 12 molecular descriptors encompass a broad range of information relevant to DDIs, providing the model with sufficiently effective and comprehensive graphical data, which significantly enhances its predictive performance.

On the other hand, Morgan fingerprints account for both the types of atoms within a chemical structure and the systematic connectivity between atoms, offering comprehensive molecular structure feature information. Consequently, both individual molecular descriptors and standalone Morgan fingerprints can yield good predictive results. Experimental results indicate that using only Morgan fingerprints can achieve an AUC value of 99.5%.

**Figure 2:**
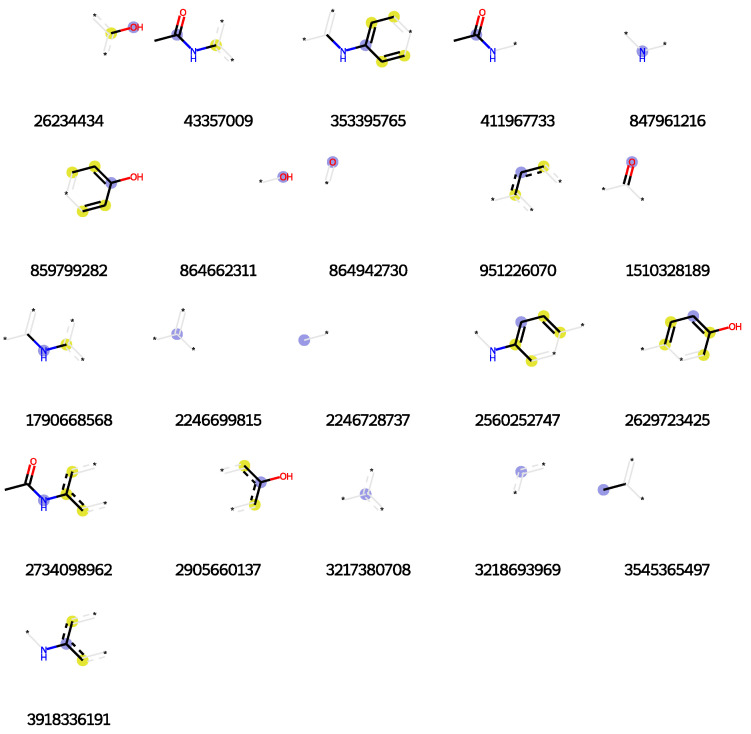
Morgan fingerprint visualization of the drug acetaminophen (blue: atomic environment center; yellow: aromatic atoms; gray: aliphatic atoms; *: there is an atom at the current position and a chemical bond exists, but it is not considered in the current fingerprint).

As shown in Figure 2, the Morgan fingerprint uses blue to represent the central atom environment, yellow for aromatic atoms, and gray for aliphatic atoms. As a hashed representation based on the atomic environment within a molecule, Morgan fingerprints capture not only the molecule’s topological structure but also its chirality. Each element in the fingerprint represents a specific structure, reflecting the chemical properties of the molecule to some extent, thereby effectively describing the chemical structure and similarity of the molecule.

In the context of pharmacokinetic DDIs, possible secondary bonding forces between drugs and the structural similarity of drugs significantly impact drug–target protein interactions. For example, bilirubin can bind to bile acids, preventing the absorption of bile acids in the digestive tract [33]. Bilirubin can also bind to other drugs (such as acetylsalicylic acid and sulfonamides), which necessitates extending the interval between taking bilirubin and other drugs as much as possible [34]. Drugs are usually transported by binding to plasma and tissue proteins, but the presence of other drugs can cause pharmacological displacement of the initial drug from tissue proteins, reducing its efficacy. For instance, the concurrent administration of warfarin and diclofenac can lead to a typical pharmacological displacement, where the increased free plasma concentration of warfarin may result in severe bleeding reactions [31]. Considering the interaction mechanisms in pharmacokinetics, such as metabolism, metabolic induction, and inhibition, further underscores the importance of chemical structure analysis in DDI prediction. Hence, the higher AUC value observed for Morgan fingerprints compared to 12 molecular descriptors might be due to Morgan fingerprints capturing the topological structures of molecules, thereby better describing their chemical features and similarities, which are crucial in DDI predictions.

When Morgan fingerprints are combined with 12 molecular descriptors, the AUC value reaches 99.9%, surpassing the 12-descriptor combination by 1.6%. Other evaluation metrics also validate this significant improvement, strongly indicating that the diversity of interpretable features enhances the model’s ability to distinguish and relate the similarities and uniqueness of chemical compounds, playing a key role in boosting model performance [35,36].

### 3.2. Model Comparison

**Figure 3:**
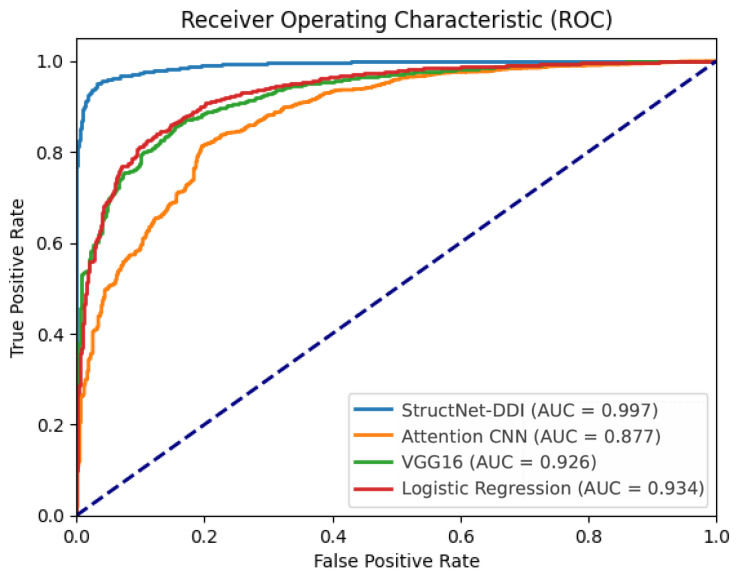
ROC curves of different models.

The performance of StructNet-DDI was compared with several methods. We used both the Morgan fingerprints and the selected 12 molecular descriptors of the samples as feature inputs and performed the corresponding feature-to-image transformations according to the model’s requirements, as referenced in the experimental results shown in Table 2 and Figure 3.

StructNet-DDI adopts a deep residual network architecture, effectively mitigating the common issues of gradient vanishing and exploding during the training of deep networks. This significantly enhances the efficiency and performance of model training. ResNet18 exhibits exceptional feature extraction capabilities, enabling it to deeply explore the complex structures and key features within the input data. For drug–drug interaction (DDI) prediction tasks, this implies that ResNet18 can more accurately identify and analyze the interaction patterns between drugs, leading to high predictive accuracy.

The experimental results demonstrate that StructNet-DDI achieved an AUC of 99.7%, with accuracy and AUPR reaching 94.4% and 99.9%, respectively, further validating the model’s effectiveness and superiority in DDI prediction. This indicates that StructNet-DDI can effectively identify interacting drug pairs, offering high reliability in practical applications.

In contrast, the Attention CNN performed poorly across various metrics. Although the attention mechanism helps focus on important regions within the data, the complexity of the Attention CNN model is higher compared to traditional CNN models, potentially requiring more training epochs and deeper parameter adjustments to show its advantages. Moreover, the suitability of the model for different application scenarios may also affect its performance in this experiment.

VGG16 showed intermediate performance compared to other models, which may be attributed to its deep architecture that enhances feature extraction capabilities. However, compared to ResNet18, which incorporates residual structures, the connections between VGG16’s convolutional layers and fully connected layers are more independent. This arrangement might reduce the reuse and sharing of features between extraction and classification tasks. Additionally, the fixed receptive field size in VGG16 limits its flexibility in learning multi-scale and deep features, potentially leading to gradient vanishing and overfitting issues.

The Logistic Regression model showed relatively poor performance on the ChCh-Miner dataset. Logistic Regression models may not be able to capture complex nonlinear features, which may play a key role in drug interactions, making deep learning models more advantageous in this regard. In terms of data representation, Logistic Regression takes feature vectors as input, whereas StructNet-DDI deals with image data. This distinction implies that the visual data produced by Morgan fingerprints and molecular descriptors more effectively capture the DDI information originating from the chemical compositions of drugs.

It is noteworthy that all four models used Morgan fingerprints and molecular descriptors as raw features, and their AUC values were all above 0.877. This further underscores the importance and indispensability of chemical structure in DDI prediction. The physicochemical properties, pharmacokinetics, topological structures, and bonding information derived from the chemical structures of drug molecules are sufficient to construct an excellent DDI prediction model.

## 4. Method

### 4.1. Molecular Structure Characteristics

In this study, we extracted Morgan fingerprints and selected molecular descriptors from the SMILES chemical structures of drug molecules as the primary features to construct the graph data for DDI prediction models.

First, the chemical structures of drug molecules were analyzed using molecular fingerprint recognition. The unique aspect of molecular fingerprint recognition lies in its ability to convert molecular structures into binary sequences, where each bit in the sequence represents the presence or absence of specific structural features at particular positions within the molecule. This encoding strategy efficiently defines the similarities and differences between molecules, laying a solid foundation for enhancing the performance of DDI prediction models. In our study, we employed the widely adopted Morgan fingerprint recognition method [36,37,38,39]. Morgan fingerprints are circular fingerprints that analyze the environment and connectivity of each atom within a chemical structure to a specified radius, encoding this information. A hashing algorithm is then used to compress the vast array of possibilities into a fixed-length fingerprint sequence. Essentially, Morgan fingerprints provide a systematic exploration of atomic types and molecular connectivity within a structure.

Additionally, we utilized molecular descriptors, which are commonly used feature extraction tools in DDI and drug–target interaction studies based on molecular structure characteristics. Molecular descriptors [40] can accurately quantify the properties of molecules, revealing their structural features, chemical and biological properties, and other multidimensional information. These descriptors can be directly calculated from molecular structures using computational chemistry software or obtained through more complex quantum chemistry calculations. To ensure the effectiveness of the model, we performed a selection process on the molecular descriptors, retaining only those strongly correlated with DDI prediction.

Ultimately, the extracted Morgan fingerprints and selected molecular descriptor features were used to construct the graph data for the prediction model, aiming to improve the model’s accuracy and robustness.

### 4.2. ResNet18 Architecture

In terms of network layer design, our prediction model adheres to the classical ResNet18 structure, incorporating convolutional layers, batch normalization layers, activation functions, and residual connections.

**Figure 4:**
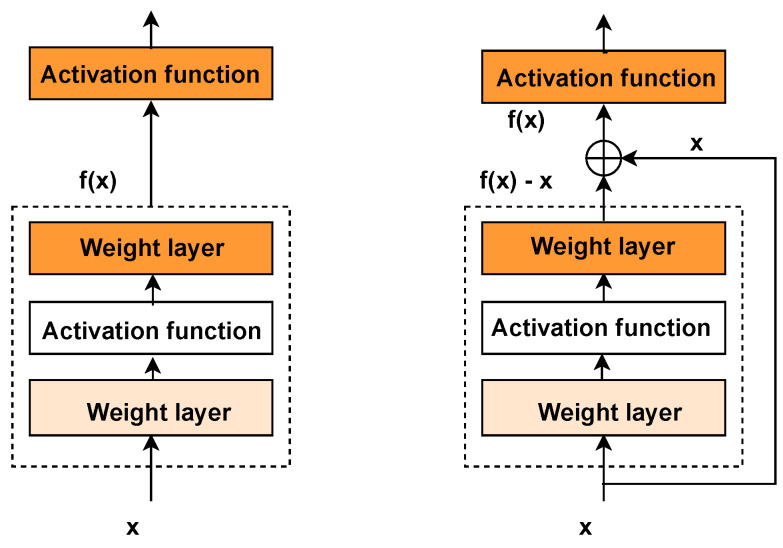
Normal block (**left**) and residual block (**right**).

For a neural network, increasing the number of layers generally allows the network to perform more complex feature extraction, which theoretically leads to better outcomes. However, this also raises the risk of problems such as vanishing or exploding gradients as the network depth increases. ResNet was introduced to address these deep model issues, with residual learning easing the optimization by bridging the gap between the input and the target mapping. If the desired mapping is denoted by (H(x)) (i.e., the original function), and the feature mapping output from the previous layer (see Figure 4) is (x) (identity function via skip connection), then the problem can be reformulated as learning the residual function (F(x)=H(x)−x). If the function being learned by the network degrades such that the primary feature is (F(x)=0), then (H(x)=x), indicating that the output equals the input. In practice, (F(x)) is unlikely to be zero, but focusing on optimizing the residual value simplifies the solution. By adopting ResNet, updating the weights for the (F(x)) part allows for seamless adaptation to new features learned. The formulation is expressed as

(1) $$y_{1}=h\left(x_{1}\right)+F\left(x_{1},W_{1}\right)$$

(2) $$x_{l+1}=f\left(y_{l}\right)$$

where $\left(h\left(x_{1}\right)\right)$ represents the input and output of the residual unit, (F) is the learned residual, $\left(W_{1}\right)$ denotes the identity mapping, and (f) is the ReLU activation function. Thus, the features learned from layer 1 to layer (L) are

(3) $$x_{L}=x_{1}+\sum_{l=1}^{L}F\left(x_{l},W_{l}\right)$$

The residual block [41] (see Figure 4) is the fundamental building block of ResNet, with its core idea revolving around the introduction of skip connections, allowing the network to learn residuals directly, thereby facilitating more efficient training. Specifically, the residual block consists of two convolutional layers with the same number of output channels, each followed by a batch normalization layer and a ReLU activation function, designed to learn feature representations from the input data. A skip connection is introduced between these two convolutional layers, enabling the input to be directly added before the final ReLU activation function, thereby allowing the network to learn the residual, i.e., the difference between the input and the desired output, rather than directly learning the output itself.

**Figure 5:**
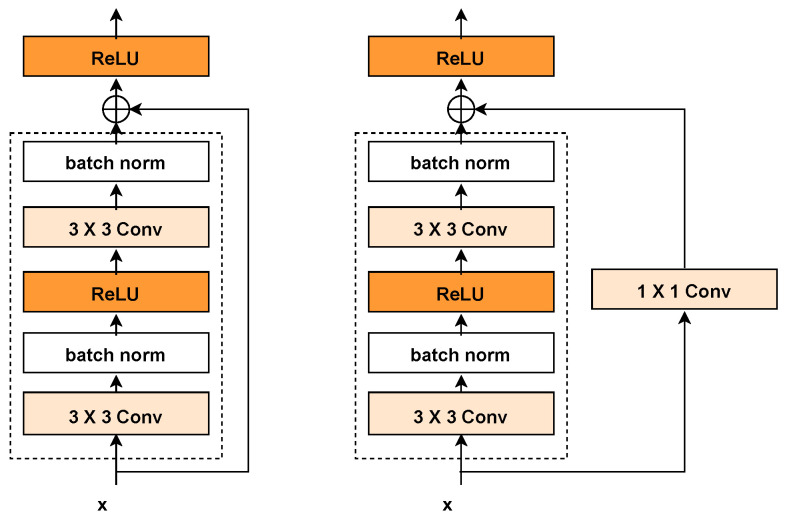
Residual block of 1 × 1 convolutional layer.

To implement the skip connection, an additional 1 × 1 convolutional layer (see Figure 5) is typically introduced. This layer adjusts the number of channels and the resolution of the input to match the output of the second convolutional layer. The combination of dual-layer convolution, the strategic skip connection, and the dimensional alignment via the 1 × 1 convolution forms the innovative design of the residual block. This design not only maintains the simplicity of the network structure but also significantly enhances the feature learning capability, thereby improving both the training efficiency and performance of the model.

In this experiment, to adapt the model for the binary classification task of predicting drug–drug interactions, we made specific modifications to the final part of the ResNet18 model. Initially, we loaded the pre-trained ResNet18 model and removed its original fully connected layer. To suit the binary classification task, we replaced it with a linear layer having two output nodes designed to predict the relationship between two input images, effectively classifying them into one of the two categories.

**Figure 6:**
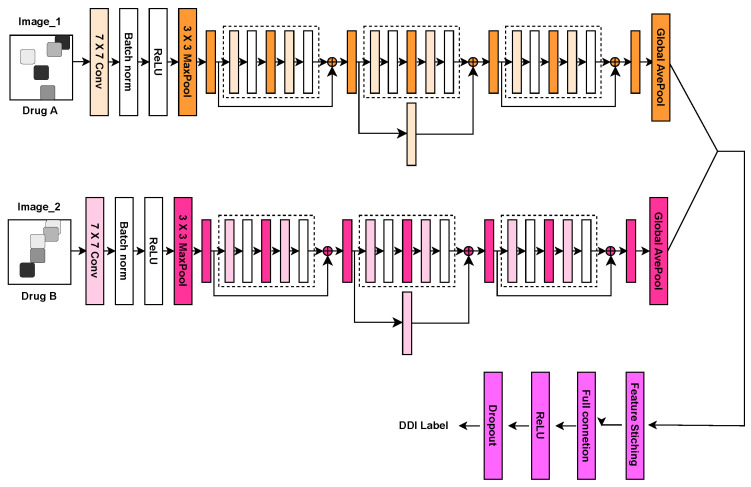
StructNet-DDI prediction model flow chart.

During forward propagation, the two input images were passed through the ResNet18 model separately to extract features, generating corresponding feature vectors. These two feature vectors were then concatenated along the channel dimension, forming a new combined feature vector that encapsulates information from both images. This combined feature vector was subsequently passed through a fully connected layer, followed by an activation function. To further prevent gradient explosion and enhance the model’s generalization capability, a Dropout layer was introduced for regularization. Finally, the model outputs a binary classification result, represented as a vector with two nodes, each corresponding to a category. In this study, one node represents the “interaction” category, while the other node represents the “non-interaction” category. The complete model flow is illustrated in Figure 6.

During the model training phase, the label information from the training dataset is utilized to guide the model in establishing an accurate classification decision model. This process involves several key steps: First, the model’s predicted outputs are compared with the actual classification labels from the validation set, and a loss metric is computed to evaluate the model’s performance. A commonly used loss function is the cross-entropy loss, which quantifies the difference between the predicted outputs and the actual labels. Next, optimization strategies, such as Stochastic Gradient Descent (SGD) or the Adam optimizer, are applied to adjust the model’s parameters through backpropagation, aiming to minimize the loss function and progressively improve the model’s classification capability. Throughout each training iteration, the model continuously learns the features of the data and optimizes its classification decisions until optimal performance is achieved.

## 5. Experiments

### 5.1. Datasets

The datasets used in this study were obtained from the research conducted by Wang et al. [42], comprising three datasets: ZhangDDI, ChCh-Miner, and DeepDDI. These datasets represent small, medium, and large scales, respectively, encompassing various important details, such as the number of drugs, DDI links, and additional key information.

ZhangDDI [6] Dataset: This dataset includes 548 drugs and 48,548 paired DDI links, along with multiple similarity metrics between these drug pairs. ChCh-Miner [43] Dataset: This dataset contains 1514 drugs and 48,514 DDI links, though it lacks some similarity information. DeepDDI [44] Dataset: Extracted from DrugBank, this dataset comprises 192,284 paired DDIs along with multi-drug side-effect information [42]. These datasets have been preprocessed to remove data items with SMILES strings that could not be converted into molecular graphs, such as SMILES strings with outdated formats or erroneous characters in DrugBank.

For this study, the ChCh-Miner dataset was selected due to its moderate size, making it well suited as a training set for initial experiments to validate and test the model’s effectiveness. To facilitate subsequent modeling and evaluation, we employed a 6:2:2 ratio to partition the dataset into training, validation, and test sets, respectively. This strategic division allows for robust model training while providing sufficient data for both validation during the development phase and an unbiased evaluation of the final model performance. The training set, comprising 60% of the data, enables the model to learn from a substantial portion of the available examples. The validation set, consisting of 20% of the data, serves to fine-tune hyperparameters and prevent overfitting. The remaining 20%, allocated to the test set, provides an independent assessment of the model’s generalization capabilities on unseen data.

In this study, we used the ChChDDI dataset to construct our DDI prediction model. It is important to note that the ChChDDI dataset only provides information on the potential for interactions between drugs, without including DMPK (Drug Metabolism and Pharmacokinetics) parameters such as drug doses or concentrations in the body. However, in practical DDI prediction, these details are crucial for assessing the intensity and clinical significance of interactions. Due to the lack of dose and concentration information, our model primarily predicts the probability of potential interactions between drugs but cannot quantify the extent of interactions or the risks at different dosage levels. This limits the model’s applicability in clinical settings, especially when dose adjustments and personalized therapy are required.

### 5.2. Acquisition of Molecular Structure Features

For each drug molecule, this study employs SMILES as the standard format for encoding chemical structure information. Utilizing functions from the RDKit library [45], SMILES strings were converted into molecular fingerprints. A custom function was developed to transform these abstract molecular data into image representations. The specific steps are described below.

Conversion of SMILES to Molecular Objects: Two SMILES strings and their corresponding labels are extracted from the dataset, and then the SMILES strings are converted into molecular objects using RDKit version 2023.09.6.

Generation of Molecular Fingerprints: RDKit is used to generate molecular fingerprints, such as Morgan fingerprints (for example, the visualization of acetaminophen’s Morgan fingerprint is shown in Figure 2) or molecular descriptors. The parameters for Morgan fingerprints are set to a length of 2048, with a radius of 2, using the GetMorganFingerprintAsBitVect function to produce hashed features.

Feature Merging and Conversion: The generated molecular fingerprints or descriptors are merged and converted into NumPy arrays, resulting in the final feature vector. Simultaneously, the corresponding labels are converted into long integer tensors for subsequent model training.

**Figure 7:**
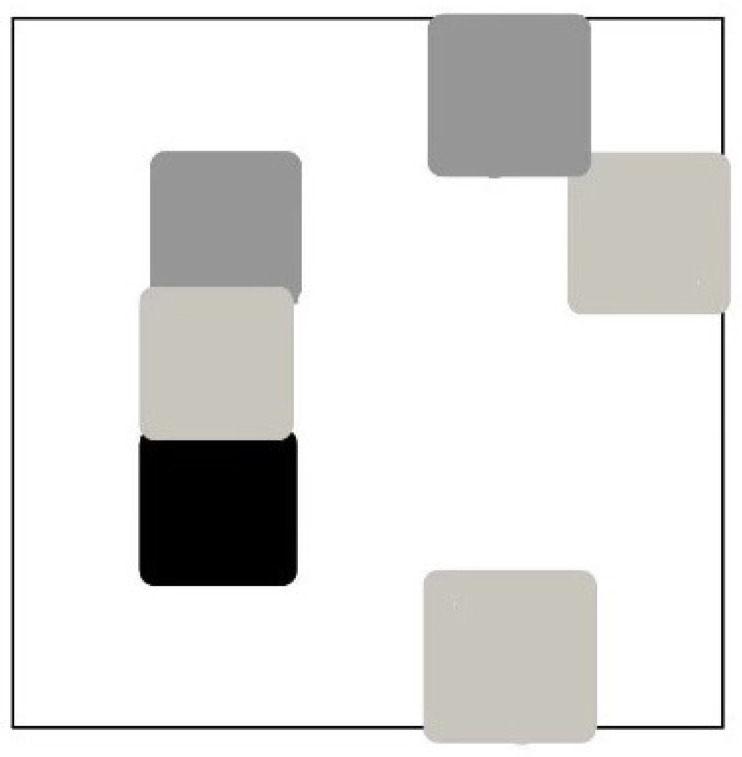
Mapping of drug molecular structure characteristics.

Feature-to-Image Conversion: The FeatureToImage function is employed to convert the molecular feature arrays into image representations. During this process, the features are mapped onto a blank image with a white background, where each feature corresponds to a point on the image (as shown in Figure 7), with the point’s color determined by the feature’s grayscale value.

Image Processing and Normalization: The generated images are resized to a uniform size and converted into tensor format. The images are then normalized to ensure consistency in the data.

Image Concatenation and Fully Connected Layer: The two generated images are concatenated along the channel dimension, resulting in a 2 × 512-dimensional feature map. Finally, a fully connected layer transforms this into a 1024-dimensional feature vector for use by the model.

In this study, to comprehensively assess the characteristics of drug molecules, we considered various aspects of their physicochemical properties, topological structures, and pharmacokinetic information. Specifically, we selected the following 12 molecular descriptors: molecular weight, logP value, relative molecular mass, the number of rotatable bonds, the number of hydrogen bond donors, the number of hydrogen bond acceptors, the number of aromatic rings, the proportion of sp3-hybridized carbons, the number of nitrogen atoms, the number of oxygen atoms, the Fereyberling index, the topological polar surface area (TPSA), and the number of free radicals. These molecular descriptors play a crucial role in evaluating feature richness and relevance, and they are instrumental in constructing the model and conducting drug–drug interaction (DDI) prediction studies [46].

### 5.3. Evaluation Metrics

Key metrics for evaluating the performance of a classification model include precision, recall, F1 Score, AUPR, AUC, and accuracy. Below is a detailed explanation of these metrics:

Precision: Precision reflects the proportion of true positive samples among those identified as positive by the model. A high precision indicates a low false positive rate, meaning the model is effective in minimizing incorrect positive predictions.

Recall: Recall describes the proportion of actual positive samples that are correctly identified by the model. A high recall indicates that the model successfully identifies most of the positive cases, thereby reducing the number of false negatives.

F1: The F1 Score is the harmonic mean of precision and recall, providing a balanced evaluation between the two. When there is a trade-off between precision and recall, the F1 Score offers a more comprehensive assessment of the model’s overall performance.

AUPR (Area Under the Precision–Recall Curve): AUPR measures the area under the precision–recall curve. When AUPR approaches 1, it indicates that the model performs well in situations with class imbalance. AUPR is particularly useful in scenarios where the ratio of positive to negative samples is significantly skewed.

AUC (Area Under the ROC Curve): AUC, representing the area under the ROC curve, is an important metric for evaluating the performance of a classification model. It assesses the model’s ability to distinguish between true positive and false positive rates at various thresholds. The higher the AUC, the better the model’s discriminative power.

Accuracy: Accuracy refers to the proportion of correctly classified samples out of the total number of samples. It is a comprehensive metric that provides an overall measure of the model’s classification performance.

### 5.4. Comparisons

To thoroughly evaluate the reliability of StructNet-DDI as a predictive model, this study compares the performance of StructNet-DDI with the following methods:

Attention CNN: A convolutional neural network optimized with an attention mechanism, which enhances performance by focusing on important features.

VGG16: VGG16 is a classical deep convolutional neural network consisting of 6 modules, with a total of 16 layers, including 13 convolutional layers, 5 pooling layers, and 3 fully connected layers. This model leverages repeated convolution operations to capture deep features of the input images.

Logistic Regression: Logistic Regression is a supervised learning method that trains a model based on a given set of data (training set) and then classifies a given set or multiple sets of data (test set). In this study, Logistic Regression is used to classify drug molecule feature vectors.

In the comparative experiments, StructNet-DDI, Attention CNN, and VGG16 all took images processed by the FeatureToImage method as input and utilized deep learning models for image classification. Logistic Regression, on the other hand, used feature vectors derived from SMILES strings for prediction. The feature vectors for all models were composed of Morgan fingerprints combined with 12 molecular descriptors, ensuring a fair comparison across different models based on the same initial features.

## 6. Conclusions

In this study, we proposed a method for DDI prediction based on the SMILES chemical structures of drug molecules. By extracting Morgan fingerprints and specific molecular descriptors, these features were transformed into raw graphical features for use in the StructNet-DDI model. The experimental results demonstrated that the basic molecular structure information and pharmacokinetic data derived from the chemical structures of drug molecules are crucial features for DDI prediction. These features provide a certain level of interpretability regarding the mechanisms of pharmacokinetic drug–drug interactions, making them sufficient for constructing high-quality raw features for the model.

The modified StructNet-DDI prediction model adopted a deep residual network architecture and incorporated regularization techniques, effectively addressing common issues such as gradient vanishing and exploding in deep network training. The model showed excellent predictive performance. Its molecular structure features are easy to construct, and the StructNet-DDI model based on graphical structures can effectively capture key features within molecular structures. Overall, it is a simple, effective, and high-performing DDI prediction model.

Furthermore, we have addressed the interpretability of our model. By visualizing the Morgan fingerprints and molecular descriptors, we are able to partially understand the model’s decision-making process. However, we acknowledge that the current level of interpretability is not sufficiently in-depth. In future work, we plan to visualize and quantitatively analyze the weights within the model’s network to more deeply reveal the impact of each feature on the prediction results. This will further enhance the model’s interpretability and performance while also promoting its greater role in drug safety evaluation and personalized therapy.

## Figures and Tables

**Table 1:** Molecular feature ablation experiments.

	Pre	Rec	F1	AUPR	AUC	Acc
3_Descriptors	0.944	0.778	0.853	0.952	0.761	0.763
6_Descriptors	0.956	0.859	0.905	0.969	0.835	0.841
12_Descriptors	0.991	0.918	0.953	0.997	0.979	0.920
Morgan	0.999	0.928	0.962	0.999	0.995	0.936
Morgan + 12_Descriptors	0.999	0.927	0.962	0.999	0.996	0.935

**Table 2:** Model performance comparison.

Model	Pre	Rec	F1	AUPR	AUC	Acc
StructNet-DDI	0.999	0.937	0.967	0.999	0.997	0.944
Attention CNN	0.969	0.773	0.861	0.979	0.877	0.778
VGG16	0.978	0.841	0.904	0.989	0.926	0.842
Logistic Regression	0.998	0.885	0.938	0.992	0.934	0.896

## Data Availability

Data were obtained from https://github.com/isjakewong/MIRACLE (accessed on 1 June 2023).

## References

[1] Lu D.Y., Lu T.R., Yarla N.S., Wu H.Y., Xu B., Ding J., Zhu H. (2017). Drug combination in clinical cancer treatments. Rev. Recent Clin. Trials.

[2] Fisusi F.A., Akala E.O. (2019). Drug combinations in breast cancer therapy. Pharm. Nanotechnol..

[3] Chee B.W., Berlin R., Schatz B. Predicting adverse drug events from personal health messages. Proceedings of the AMIA Annual Symposium Proceedings.

[4] Budnitz D.S., Pollock D.A., Weidenbach K.N., Mendelsohn A.B., Schroeder T.J., Annest J.L. (2006). National surveillance of emergency department visits for outpatient adverse drug events. JAMA.

[5] Lin S., Zhang G., Wei D.Q., Xiong Y. (2022). DeepPSE: Prediction of polypharmacy side effects by fusing deep representation of drug pairs and attention mechanism. Comput. Biol. Med..

[6] Zhang W., Chen Y., Liu F., Luo F., Tian G., Li X. (2017). Predicting potential drug-drug interactions by integrating chemical, biological, phenotypic and network data. BMC Bioinform..

[7] Cheng F., Zhao Z. (2014). Machine learning-based prediction of drug–drug interactions by integrating drug phenotypic, therapeutic, chemical, and genomic properties. J. Am. Med. Inform. Assoc..

[8] Hunta S., Yooyativong T., Aunsri N. (2018). A novel integrated action crossing method for drug-drug interaction prediction in non-communicable diseases. Comput. Methods Programs Biomed..

[9] Chen Y., Ma T., Yang X., Wang J., Song B., Zeng X. (2021). MUFFIN: Multi-scale feature fusion for drug–drug interaction prediction. Bioinformatics.

[10] Huang K., Xiao C., Hoang T., Glass L., Sun J. Caster: Predicting drug interactions with chemical substructure representation. Proceedings of the AAAI Conference on Artificial Intelligence.

[11] Deng Y., Xu X., Qiu Y., Xia J., Zhang W., Liu S. (2020). A multimodal deep learning framework for predicting drug–drug interaction events. Bioinformatics.

[12] Nyamabo A.K., Yu H., Liu Z., Shi J.Y. (2022). Drug–drug interaction prediction with learnable size-adaptive molecular substructures. Briefings Bioinform..

[13] Kim S., Chen J., Cheng T., Gindulyte A., He J., He S., Li Q., Shoemaker B.A., Thiessen P.A., Yu B. (2021). PubChem in 2021: New data content and improved web interfaces. Nucleic Acids Res..

[14] Kuhn M., Campillos M., Letunic I., Jensen L.J., Bork P. (2010). A side effect resource to capture phenotypic effects of drugs. Mol. Syst. Biol..

[15] Li P., Huang C., Fu Y., Wang J., Wu Z., Ru J., Zheng C., Guo Z., Chen X., Zhou W. (2015). Large-scale exploration and analysis of drug combinations. Bioinformatics.

[16] Dang L.H., Dung N.T., Quang L.X., Hung L.Q., Le N.H., Le N.T.N., Diem N.T., Nga N.T.T., Hung S.H., Le N.Q.K. (2021). Machine learning-based prediction of drug-drug interactions for histamine antagonist using hybrid chemical features. Cells.

[17] Takeda T., Hao M., Cheng T., Bryant S.H., Wang Y. (2017). Predicting drug–drug interactions through drug structural similarities and interaction networks incorporating pharmacokinetics and pharmacodynamics knowledge. J. Cheminform..

[18] Zhang Y., Yao Q., Yue L., Wu X., Zhang Z., Lin Z., Zheng Y. (2023). Emerging drug interaction prediction enabled by a flow-based graph neural network with biomedical network. Nat. Comput. Sci..

[19] Yu H., Li K., Dong W., Song S., Gao C., Shi J. (2023). Attention-based cross domain graph neural network for prediction of drug–drug interactions. Briefings Bioinform..

[20] Yin Q., Fan R., Cao X., Liu Q., Jiang R., Zeng W. (2023). Deepdrug: A general graph-based deep learning framework for drug-drug interactions and drug-target interactions prediction. Quant. Biol..

[21] Zhang X., Wang G., Meng X., Wang S., Zhang Y., Rodriguez-Paton A., Wang J., Wang X. (2022). Molormer: A lightweight self-attention-based method focused on spatial structure of molecular graph for drug–drug interactions prediction. Briefings Bioinform..

[22] Jiang M., Liu G., Zhao B., Su Y., Jin W. (2024). Relation-aware graph structure embedding with co-contrastive learning for drug–drug interaction prediction. Neurocomputing.

[23] Jin B., Yang H., Xiao C., Zhang P., Wei X., Wang F. Multitask dyadic prediction and its application in prediction of adverse drug-drug interaction. Proceedings of the AAAI Conference on Artificial Intelligence.

[24] Zhou Y., Hou Y., Shen J., Huang Y., Martin W., Cheng F. (2020). Network-based drug repurposing for novel coronavirus 2019-nCoV/SARS-CoV-2. Cell Discov..

[25] Liang Y. (2023). DDI-SSL: Drug–drug interaction prediction based on substructure signature learning. Appl. Sci..

[26] Elton D.C., Boukouvalas Z., Fuge M.D., Chung P.W. (2019). Deep learning for molecular design—A review of the state of the art. Mol. Syst. Des. Eng..

[27] O’Boyle N., Dalke A. (2018). DeepSMILES: An Adaptation of SMILES for Use in Machine-Learning of Chemical Structures. https://chemrxiv.org/engage/chemrxiv/article-details/60c73ed6567dfe7e5fec388d.

[28] Xue D., Zhang H., Xiao D., Gong Y., Chuai G., Sun Y., Tian H., Wu H., Li Y., Liu Q. (2020). X-MOL: Large-scale pre-training for molecular understanding and diverse molecular analysis. bioRxiv.

[29] Zhong Y., Zheng H., Chen X., Zhao Y., Gao T., Dong H., Luo H., Weng Z. (2023). DDI-GCN: Drug-drug interaction prediction via explainable graph convolutional networks. Artif. Intell. Med..

[30] Ma J., Sheridan R.P., Liaw A., Dahl G.E., Svetnik V. (2015). Deep neural nets as a method for quantitative structure–activity relationships. J. Chem. Inf. Model..

[31] Palleria C., Di Paolo A., Giofrè C., Caglioti C., Leuzzi G., Siniscalchi A., De Sarro G., Gallelli L. (2013). Pharmacokinetic drug-drug interaction and their implication in clinical management. J. Res. Med Sci. Off. J. Isfahan Univ. Med Sci..

[32] Niu J., Straubinger R.M., Mager D.E. (2019). Pharmacodynamic drug–drug interactions. Clin. Pharmacol. Ther..

[33] Scaldaferri F., Pizzoferrato M., Ponziani F.R., Gasbarrini G., Gasbarrini A. (2013). Use and indications of cholestyramine and bile acid sequestrants. Intern. Emerg. Med..

[34] Phillips W.A., Ratchford J.M., Schultz J.R. (1976). Effects of colestipol hydrochloride on drug absorption in the rat II. J. Pharm. Sci..

[35] Hosmer D.W., Lemeshow S., Sturdivant R.X. (2013). Applied Logistic Regression.

[36] Rogers D., Hahn M. (2010). Extended-connectivity fingerprints. J. Chem. Inf. Model..

[37] Pham T., Ghafoor M., Grañana-Castillo S., Marzolini C., Gibbons S., Khoo S., Chiong J., Wang D., Siccardi M. (2024). DeepARV: Ensemble deep learning to predict drug-drug interaction of clinical relevance with antiretroviral therapy. NPJ Syst. Biol. Appl..

[38] Shtar G., Solomon A., Mazuz E., Rokach L., Shapira B. (2023). A simplified similarity-based approach for drug-drug interaction prediction. PLoS ONE.

[39] Wang G., Feng H., Cao C. (2024). BiRNN-DDI: A Drug-Drug Interaction Event Type Prediction Model Based on Bidirectional Recurrent Neural Network and Graph2Seq Representation. J. Comput. Biol..

[40] An X., Chen X., Yi D., Li H., Guan Y. (2022). Representation of molecules for drug response prediction. Briefings Bioinform..

[41] Targ S., Almeida D., Lyman K. (2016). Resnet in resnet: Generalizing residual architectures. arXiv.

[42] Wang Y., Min Y., Chen X., Wu J. Multi-view graph contrastive representation learning for drug-drug interaction prediction. Proceedings of the Web Conference 2021.

[43] Zitnik M., Rok Sosič S.M., Leskovec J. (2018). BioSNAP Datasets: Stanford Biomedical Network Dataset Collection. http://snap.stanford.edu/biodata.

[44] Ryu J.Y., Kim H.U., Lee S.Y. (2018). Deep learning improves prediction of drug–drug and drug–food interactions. Proc. Natl. Acad. Sci. USA.

[45] Landrum G. (2013). RDKit: A software suite for cheminformatics, computational chemistry, and predictive modeling. Greg Landrum.

[46] Lenselink E.B., Ten Dijke N., Bongers B., Papadatos G., Van Vlijmen H.W., Kowalczyk W., IJzerman A.P., Van Westen G.J. (2017). Beyond the hype: Deep neural networks outperform established methods using a ChEMBL bioactivity benchmark set. J. Cheminform..

